# Evaluation of a nitrous oxide mobile destruction unit to reduce occupational exposure in a burns department

**DOI:** 10.1111/anae.70221

**Published:** 2026-04-28

**Authors:** Christelle Machon, Anthony Chaverot, Amélie Massardier‐Pilonchery, Vincent Reneric, François Parant

**Affiliations:** ^1^ Hospices Civils de Lyon Lyon France

Entonox^®^ (BOC Limited, Woking, UK) is used commonly for analgesia due to its rapid onset and ease of administration [1]. However, nitrous oxide is a potent greenhouse gas with a global warming potential [2]. Beyond its environmental footprint, chronic occupational exposure to nitrous oxide is associated with potential health risks for healthcare professionals [3]. In France, the Agence nationale de sécurité sanitaire de l'alimentation, de l'environnement et du travail (ANSES) has proposed strict occupational threshold limit values. These are a time‐weighted average of 25 parts per million (ppm) for >8 h and a short‐term exposure limit 125 ppm for >15 min. Despite organisational measures, staff training and the use of on‐demand valves, recent metrological campaigns indicate that these strategies are often insufficient to maintain ambient nitrous oxide concentrations below recommended thresholds [4]. We aimed to evaluate the efficacy of a nitrous oxide mobile destruction unit to reduce occupational exposure during painful procedures in a clinical setting.

Evaluations were conducted in November 2024 at the Burn Center Pierre Colson (Hospices Civils de Lyon, Lyon, France) in a day unit. Nitrous oxide concentrations were measured in the breathing zone of nursing staff during Entonox administration of 21 distinct cases, including dressing changes and debridement. Twelve cases were performed with the mobile destruction unit active, while nine served as controls without the device. The device used was a Medclair Mobile Destruction Unit (Medclair Invest, Stockholm, Sweden), which operates on the principle of thermal catalytic cracking. This process decomposes exhaled nitrous oxide into its primary components, nitrogen and oxygen, which are safe for humans and are non‐greenhouse gases. Ambient nitrous oxide concentrations were monitored in semi‐real‐time (one measurement per minute) using a portable Geotech G200 infrared analyser (Geotech, Maidstone, UK) with a measurement range of 0–1000 ppm. The primary performance indicator was the proportion of measurements remaining below the short‐term threshold as recommended by ANSES.

We collected 183‐point measurements during 21 sessions, including 97 (53%) with the mobile destruction unit active. The median (IQR [range]) duration of Entonox administration was 7 (6–12 [3–20]) min. Without the mobile destruction unit, the nitrous oxide ambient concentration was 186 (73–458 [0–1000]) ppm (Table 1). Under these control conditions, only 28 (33%) measurements and one (11%) case were below the 125 ppm threshold. In contrast, the use of the mobile destruction unit led to a reduction of nitrous oxide ambient levels, with a median (IQR [range]) concentration of 12 (1–27 [0–918]) ppm (Table 1). The mobile destruction unit allowed 95 (95%) nitrous oxide measurements and nine (75%) cases to be maintained below the 125 ppm threshold. Values >500 ppm observed when the mobile destruction unit was used were due to nitrous oxide leak because the mask was moved (918 ppm) or around the mouth because the patient was tense due to pain (537 ppm).

**Table 1:** Ambient nitrous oxide levels measured in the breathing zone of nursing staff during Entonox administration with and without the use of a nitrous oxide mobile destruction unit. Values are median (IQR [range]).

Use of mobile destruction unit	Entonox duration of use; min	Nitrous oxide; ppm
Yes	15	13 (3–28 [0–71])
Yes	6	5 (3–13 [2–25])
Yes	7	20 (20–23 [1–31])
Yes	4	13 (6–19 [1–24])
Yes	8	3 (1–6 [0–8])
Yes	13	55 (46–96 [9–918])
Yes	7	15 (12–19 [12–26])
Yes	4	46 (20–85 [0–146])
Yes	12	8 (2–18 [0–39])
Yes	6	0 (0–3 [0–40])
Yes	9	0 (0–0 [0–0])
Yes	6	88 (24–340 [0–537])
No	13	190 (52–519 [0–1000])
No	6	154 (91–166 [0–177])
No	5	375 (98–1000 [0–1000])
No	3	20 (13–44 [6–68])
No	16	306 (92–449 [7–1000])
No	6	120 (53–148 [31–183])
No	8	123 (33–165 [14–321])
No	9	172 (25–329 [0–712])
No	20	485 (249–560 [0–687])

Our results are consistent with recent literature reported previously in obstetric settings, which have shown that cracking technology can reduce ambient nitrous oxide levels by 71–81% in real‐world clinical environments [3, 4]. Here, we have shown the high efficacy of the mobile destruction unit to reduce nitrous oxide ambient levels in a burns centre during short‐term cases. Indeed, the median values of nitrous oxide ambient levels were reduced by 90% with the use of the mobile destruction unit. The primary advantage is its dual benefit, as it protects staff from occupational exposure while simultaneously reducing the environmental impact of medical gas use. This distinguishes it from central scavenging systems or ‘double mask’ suction systems, which typically collect nitrous oxide and discharge it into the external atmosphere without any treatment. The mobile destruction unit also has good ergonomics. However, its efficacy is limited by the patient–device interface. Any leakage around the facemask or mouthpiece, often caused by patient discomfort, poor mask fit or nasal exhalation, results in immediate spikes in ambient air that bypass the mobile destruction unit. Staff feedback indicated that although the device is effective, its implementation requires ongoing support to ensure proper mask fit and the absence of leaks during nitrous oxide administration.

The mobile destruction unit can be considered as a flexible and highly effective solution for reducing occupational exposition to nitrous oxide, especially in poorly ventilated rooms or rooms without openings to the outside, if it is accompanied by rigorous staff training and strict adherence to best procedural practices to reduce sources of nitrous oxide leakage. It therefore represents an effective solution for complying with short‐term nitrous oxide exposure limits.
